# Enhanced FFT–Root–MUSIC Algorithm Based on Signal Reconstruction via CEEMD–SVD for Joint Range and Velocity Estimation for FMCW Radar

**DOI:** 10.3390/s24248000

**Published:** 2024-12-14

**Authors:** Jiaxin Cao, Huiyue Yi, Wuxiong Zhang, Hui Xu

**Affiliations:** 1 Key Laboratory of Science and Technology on Micro-System, Shanghai Institute of Microsystem and Information Technology Chinese Academy of Sciences, Shanghai 200050, China; caojiaxin22@mail.sim.ac.cn (J.C.); wuxiong.zhang@mail.sim.ac.cn (W.Z.); hui.xu@mail.sim.ac.cn (H.X.); 2 University of Chinese Academy of Sciences, Beijing 100049, China

**Keywords:** FMCW radar, CEEMD, SVD, FFT-Root-MUSIC algorithm, joint range–velocity estimation

## Abstract

Frequency-modulated continuous-wave (FMCW) radar is used to extract range and velocity information from the beat signal. However, the traditional joint range–velocity estimation algorithms often experience significant performances degradation under low signal-to-noise ratio (SNR) conditions. To address this issue, this paper proposes a novel approach utilizing the complementary ensemble empirical mode decomposition (CEEMD) combined with singular value decomposition (SVD) to reconstruct the beat signal prior to applying the FFT-Root-MUSIC algorithm for joint range and velocity estimation. This results in a novel joint range–velocity estimation algorithm termed as the CEEMD-SVD-FFT-Root-MUSIC (CEEMD-SVD-FRM) algorithm. First, the beat signal contaminated with additive white Gaussian noise is decomposed using CEEMD, and an appropriate autocorrelation coefficient threshold is determined to select the highly correlated intrinsic mode functions (IMFs). Then, the SVD is applied to the selected highly correlated IMFs for denoising the beat signal. Subsequently, the denoised IMFs and signal residuals are combined to reconstruct the beat signal. Finally, the FFT-Root-MUSIC algorithm is applied to the reconstructed beat signal to estimate both the range and Doppler frequencies, which are then used to calculate the range and velocity estimates of the targets. The proposed CEEMD-SVD-FRM algorithm is validated though simulations and experiments, demonstrating significant improvement in the robustness and accuracy of range and velocity estimates for the FMCW radar due to the effective denoising of the reconstructed beat signal. Moreover, it substantially outperforms the traditional methods in low SNR environments.

## 1. Introduction

Traditional hydrological measurement techniques include water level measurement, flow velocity measurement, and water volume estimation. Water level and flow velocity measurements are primarily utilized in fields such as freshwater fisheries research, river hydrology studies, river navigation safety, flood and drought prevention, and comprehensive water resource management [1]. Currently, water measurement techniques predominantly rely on contact-based methods, using conventional cableways or measurement boats to gauge water levels and flow velocities. However, these methods encounter challenges such as low operational efficiency, high safety risks, significant manual measurement errors, and interference from water impurities. With advancements in society, technology, and economics, new measurement methods and signal processing technologies have continued to emerge. To meet the demands for high resolution, high precision, strong disturbance resistance, ease of installation, low maintenance costs, and measurement safety, non-contact radar measurement technology is increasingly being researched and applied for the remote monitoring of rivers, wells, sedimentation basins, and water treatment facilities [2].

Radar is a technology that employs radio waves to detect, measure, and track targets. By emitting radio electromagnetic waves and receiving the signals reflected from targets, radar can determine and locate the target’s spatial position by analyzing these reflected signals. Even under various interference conditions such as wind, rain, fog, light, humidity, and temperature, radar can still determine the angular position, range, speed, and other identifying features of targets [3]. Common radar systems are categorized into continuous wave (CW) and pulse wave systems [4], as shown in Table 1. Compared to the pulse radar, the CW radar features separate transmitting and receiving devices, allowing for simultaneous signal transmission and reception, virtually eliminating detection blind spots caused by the time gaps between transmission and reception [5]. The frequency-modulated continuous-wave (FMCW) radar provides advantages such as low-power consumption, no range blind spots, low cost, high resolution, and strong anti-interference capabilities. Consequently, the FMCW radar is extensively used in hydrology due to its high-precision measurement capabilities and low cost. The FMCW radar measures the range and velocity of targets by estimating the range and Doppler frequencies from the beat signal. The accuracy of range and velocity estimates is directly linked to the accuracy of the range and Doppler frequency estimates derived from the beat signal [6,7]. Therefore, accurately and rapidly performing joint estimation for the range and velocity of targets using the FMCW radar in complex environments has become a key focus in radar measurement research.

The FMCW radar estimates the range and velocity of targets by analyzing the frequency shifts caused by the Doppler effect and the time delays of reflected signals. The 2D-FFT algorithm [8,9,10] is a widely used method for joint range and velocity estimation in FMCW radar systems. However, the fast Fourier transform (FFT) algorithm [11,12] faces challenges such as aliasing, spectral leakage, and the fence effect, which results in substantial errors in the amplitude, frequency, and phase estimates of the detected signals. Moreover, the Fourier transform is sensitive to noise [13], especially in low signal-to-noise ratio (SNR) conditions, which limits the ability of the FFT-based method to accurately estimate the target information. Ali et al. [8] proposed a 2D-FFT algorithm for detecting extremely weak moving targets with FMCW radar, which enables accurate range–velocity pairing while providing strong anti-jamming capability, though it suffers from high complexity and poor stability. Song et al. [9] developed a 2D-FFT algorithm that addresses the range–velocity ambiguity problem by employing multiple frequency ramps, but their approach has high false-alarm rates and limited resolution. Seifallah et al. [10] combined the 2D-FFT algorithm with the Newton gradient algorithm for parameter estimation, improving spatial resolution and reducing computational complexity. Furthermore, researchers have proposed other new algorithms, such as spectrum refinement algorithms [14], ratio algorithms [15], phase difference algorithms [16], super-resolution algorithms [17], and sparse optimization algorithms [18]. These algorithms aim to overcome the limitations of existing algorithms in terms of low resolution, poor accuracy, weak robustness, and high computational complexity. To improve frequency resolution and estimation accuracy, Wen et al. [19] utilized the 2D-Unitary ESPRIT algorithm for joint range and velocity estimation in FMCW radar, but it suffers from high computational complexity. Similarly, Kim et al. [20] proposed a range–Doppler estimation method based on the FFT-MUSIC algorithm, but its performance degrades in nonlinear noisy environments. To reduce the computational complexity, Kim et al. [21] proposed two low-complexity algorithms, though their resolution and accuracy degrade in complex multi-target environments. Moussa [22] proposed a two-stage algorithm that decouples range and Doppler domains using one-dimensional searches and the CLEAN technique [23] for pairing estimated parameters of multiple targets. However, their method still encounters significant mismatch rates under low SNR conditions.

To tackle the issues of poor resolution, low-estimation accuracy, and weak robustness for range and velocity estimation of the existing algorithms in low SNR environments, this paper proposes to reconstruct the beat signal by utilizing the complementary ensemble empirical mode decomposition (CEEMD) and the singular value decomposition (SVD) to denoise the beat signal before applying the FFT-Root-MUSIC algorithm for joint range and velocity estimation based on FMCW radar, resulting in the CEEMD-SVD-FFT-Root-MUSIC (abbreviated as CEEMD-SVD-FRM) algorithm. Firstly, the empirical mode decomposition (EMD) [24] method was explored. To overcome the mode-mixing problem in EMD and achieve complete signal decomposition, the CEEMD method [25] is applied to the intermediate frequency signal (IFS), which is expressed as a discrete time-domain signal in (6). The CEEMD adds pairs of white noise with equal amplitude and opposite signs to the signal, which effectively cancels out during decomposition. This method decomposes the signal into several intrinsic mode functions (IMFs) and the residuals to reduce reconstruction errors. For the set of IMFs obtained after decomposition, those with higher correlation to the original signal contain more effective signal and noise. Therefore, the correlation coefficients are utilized to select the IMFs that have high correlation with the original signal, preserving significant effective information while suppressing noise. As the residual noise can propagate from higher-frequency IMFs to lower-frequency IMFs and affect the entire signal, the SVD method [26] is utilized for noise reduction, implementing the combined use of CEEMD and SVD. Then, new high-correlation IMFs are obtained by selecting an appropriate number of largest singular values of the SVD. After denoising, the denoised IMFs and the signal residual are reconstructed to obtain the denoised reconstructed signal. Based on this reconstructed signal, the FFT and Root-MUSIC algorithm are applied to estimate the range frequency and Doppler frequency, and then the range and velocity of the targets are obtained. Finally, the simulation experiments validate that the proposed CEEMD-SVD-FRM algorithm achieves higher resolution, estimation accuracy, and robustness in joint range and velocity estimation compared to the existing algorithms, especially in low SNR conditions.

The main contributions of this paper are as follows. Firstly, the CEEMD and SVD methods are jointly utilized to denoise and reconstruct the beat signal to mitigate the negative impact of the noise on the estimation performance of the range and velocity estimation in the FMCW radar. As the CEEMD utilizes multiple IMFs to represent the signal characteristics, it effectively avoids the limitations of FFT and handles the non-stationary and non-linear characteristics caused by target movement, multipath effects, and radar hardware or dynamic influences. Moreover, to mitigate the impact of residual noise from the CEEMD on the signal, the SVD method is employed to further denoise the signal while preserving the effective signal. By selecting an appropriate number of largest singular values of the SVD, the denoised high-correlation IMFs and residuals are reconstructed to obtain the denoised signal. Based on the reconstructed signal, the FFT and Root-MUSIC algorithms are jointly utilized to achieve joint range and velocity estimation.

The remainder of this paper is organized as follows: Section 2 describes the signal model and related works. Section 3 describes the proposed CEEMD-SVD-FRM algorithm, with a specific emphasis on the joint denoising process that integrates CEEMD and SVD method. In Section 4, both simulation and field test results are presented, with a comparative analysis against existing algorithms to highlight the performance of the proposed method. Finally, Section 5 concludes the paper, discussing the significance of the findings and suggesting potential directions for future research.

## 2. Signal Model and Related Works

In this section, the signal model is first described. Then, the traditional 2D-FFT algorithm for range and velocity estimation [9,10] and the FFT-MUSIC algorithm for joint range and velocity estimation [20] are described. Finally, a comparison between the two algorithms is presented, summarizing their advantages and disadvantages while also pointing out their limitations. Moreover, the motivation of this paper is provided.

### 2.1. Signal Model

The transmitted signal of the FMCW radar [19] can be expressed as

(1) $$s_{tx}\left(t\right)=a_{tx}\cdot exp\left\{j2\pi \left(f_{0}t+\frac{1}{2}\mu t^{2}\;\right)+\varphi_{0}\;\right\},\;0<t<T_{c}$$

where $a_{tx},\;f_{0},\;\mu ,\;\varphi_{0},\;T_{c}$ denote the amplitude gain of the transmitted signal, carrier frequency, the slope of FMCW chirp $\mu =B/T_{c}$ (where B is the chirp bandwidth and $T_{c}$ is the chirp ramp duration), and the initial phase and ramp duration of the transmitted signal, respectively.

The received signal, reflected by L targets, can be expressed as follows:

(2) $$s_{rx}(t)\;=\;\sum_{l\;=\;1}^{L}a_{rx(l)}\;exp\left\{\;j2\pi \left(f_{0}\left(\;t-\tau_{l\;}\right)+\;\frac{1}{2}\mu {\left(\;t\;-\;\tau_{l\;}\right)}^{2}\right)\;+\;\varphi_{1(l)}\right\}+\;z(t)$$

where $a_{rx},\;\tau_{l\;},\;\varphi_{1(l)}$, z(t) denotes the amplitude gain of the received signal, the time delay of the l-th target, the phase of the l-th target and the noise component, respectively.

By mixing the transmitted and received signals to obtain the in-phase component [27], and passing it through a low-pass filter, combining the in-phase component with the quadrature component results in the complex signal, also known as the IFS x(t) [28]. Therefore, the IFS for L targets is

(3) $$x(t)\;=\sum_{l=1}^{L}a_{mix(l)}\;exp\left\{\;j2\pi \left(\;f_{0}\tau_{l\;}+\mu \tau_{l}t+\frac{1}{2}\mu {\tau_{l}}^{2}\right)+\varphi_{rx(l)}\;\right\}\;+z(t)$$

### 2.2. Related Works

#### 2.2.1. 2D-FFT Algorithm Joint Range–Velocity Estimation

**Figure 1:**
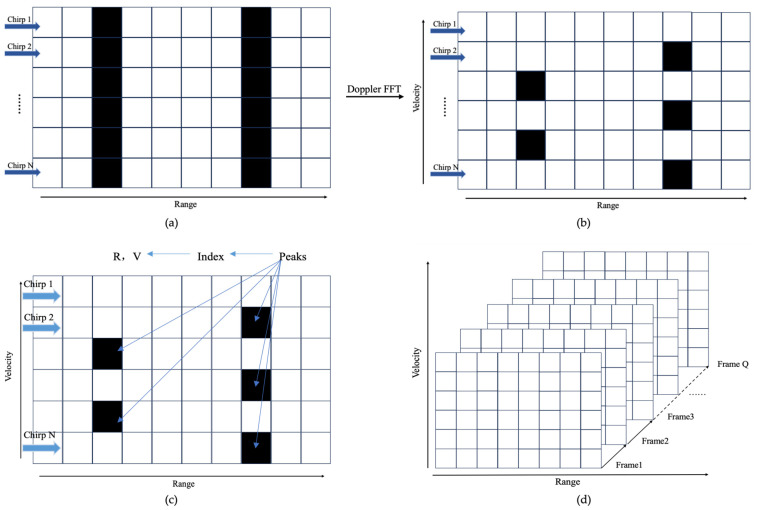
2D-FFT processing flow. (**a**) Range FFT; (**b**) Doppler FFT; (**c**) finding the spectral peaks and obtaining the corresponding index values; (**d**) processing each frame.

Assuming each frame consists of M chirp cycles, with each chirp containing N sampling points, where *n* denotes the n-th sampling point of the chirp signal and m denotes the m-th chirp signal in the sequence, as shown in Figure 1. The 2D-FFT algorithm [29] involves applying FFT separately on the fast-time and slow-time dimensions. Each frame undergoes a two-dimensional Fourier transform of N points across M periods of chirps, enabling the extraction of the range and velocity information of the measured target. Firstly, a range FFT is performed on the digitized samples corresponding to each chirp, as shown in Figure 1a. The output results are stored in a matrix with continuous rows. Once all individual chirps in a frame have been received and processed by the processor, it begins performing FFT on the chirp sequence (Doppler FFT), as depicted in Figure 1b. The combined operation of range FFT (line by line) and Doppler FFT (column by column) can be viewed as a 2D FFT of the digitized sampling points corresponding to each frame. The peak positions in the spectrum correspond to the range and velocity of the target in front of the radar, as illustrated in Figure 1c. By using the index values corresponding to the spectral peaks, the target’s range and velocity can then be calculated, and each frame is processed accordingly, as demonstrated in Figure 1d.

Consider L targets moving with a radial velocity relative to the radar $v_{0l}(l=1,\;2,\ldots ,L)$, as shown in Figure 1. In this figure, the 2D-FFT algorithm can detect five targets (L = 5). For an FMCW radar signal, the radial velocity of a target is considered positive when the target is moving away from the radar. This is due to the fact that a positive Doppler shift is observed when the target is receding. Assuming that the target’s motion only occurs between frequency sweeps and remains stationary within a single sweep, the time delay corresponding to the l-th target can be expressed as

(4) $$\tau_{l}=\frac{2\left(\;R_{0l}+v_{0l}\cdot {∆t}_{m}\right)}{c}$$

where $R_{0l}$ is the initial position of the l-th target, ${∆t}_{m}=mT_{l}$, $T_{l}$ is the pulse repetition interval, i.e., $T_{l}=T_{c}+T_{z}$, $T_{z}$ is the time interval between the chirps, and $v_{0l}$ is the initial velocity of the l-th target.

Then, the time ${∆t}_{m}(n)$ corresponding to the n-th sampling point of the m-th chirp signal can be expressed as

(5) $${∆t}_{m}(n)=m\;\cdot T_{l}+\frac{n}{f_{s}},\;-\frac{N}{2}\;\leq \;n\;\leq \;\frac{N}{2}$$

Substituting (4) and (5) into (3), the discrete time-domain expression for the IFS x(t) can be expressed as

(6) $$x(n,\;m)=\sum_{l=1}^{L}a_{mix\;}exp\left\{\;j4\pi \left(\frac{f_{0}R_{0l}}{c}+\frac{f_{0}v_{0l}\cdot {\;T}_{l}\cdot \;m}{c}+\frac{(\mu R_{0l}+f_{0}v_{0l})n}{{cf}_{s}}+\frac{\mu v_{0l}\cdot T_{l}\cdot \;mn}{{cf}_{s}}+\frac{\mu v_{0l}\cdot {\;n}^{2}}{{{cf}_{s}}^{2}}\right)\right\}+z(n,\;m)$$

where 0 ≤ n≤ N−1, 0 ≤ m ≤ M−1, and z(n, m) is the corresponding noise term.

Therefore, ignoring the higher-order terms and performing denoising, the range FFT is applied to (6) in the fast-time dimension to obtain the target cells in the range dimension:

(7) $$x_{r}\left(\gamma ,\;m\right)\;=\;\sum_{l=1}^{L}w_{\vartheta }\cdot exp\left\{\;j4\pi \left(\frac{f_{0}R_{0l}}{c}\;+\;\frac{f_{0}v_{0l}\cdot \;T_{l}\cdot \;m}{c}\right)\right\}\cdot f_{sinc}\left(\gamma \;-\;N\frac{2\mu R_{0l}\;+\;2f_{0}v_{0l}\;+\;2\mu v_{0l}\cdot T_{l}\cdot \;m}{{cf}_{s}}\right)$$

where γ represents the range unit, $w_{\vartheta }=a_{mix}N$, and the expression for the function $f_{sinc}$ is equal to sin⁡(πn)/sin⁡(πn/N).

Subsequently, based on the range FFT results processed in the fast-time dimension, as see in (7), performing a second FFT in the slow-time dimension yields the range–velocity expression for the measured targets:

(8) $$x_{d}(\gamma_{target},\;\vartheta )\;=\;\sum_{l=1}^{L}w_{\vartheta }\cdot {\;e\;}^{j4\pi \frac{f_{0}R_{0l}}{c}}\cdot \left(e^{\;-j\;\frac{2\pi v_{0l}}{M}\cdot \;\frac{(M-1)}{2}}\;\cdot \;f_{sinc}\left(\vartheta \;-M\frac{2f_{0}v_{0l}\cdot T_{l}}{c}\right)\right)\;*\;B(\vartheta )$$

where $\gamma_{target}\;$ represents the range unit of the target, ϑ represents the Doppler unit, $w_{\vartheta }=a_{mix}\cdot N^{2}\cdot M$,B(ϑ) represents the Fourier transform of the amplitude offset function, and ∗ represents convolution. Moreover, the convolution of the ${f\;}_{sinc}$ function and B(ϑ) in the expression does not change the position of the spectral peak of the ${f\;}_{sinc}$ function.

In summary, the expressions for the range unit and the velocity unit of the measured target can be derived from (8) [9]:

(9) $$\gamma_{l}\;=\;N\;\frac{2\mu R_{0l}+\;2f_{0}v_{0l}}{{c\;f}_{s}}\;=\;(f_{dl}\;+\;f_{rl})NT_{s}$$

(10) $$\vartheta_{l}=M\;\frac{2{\;f}_{0}v_{0l}\;\cdot T_{l}}{c}=M\cdot \;T_{l}\cdot \;f_{dl}$$

where $f_{dl}\;=\;2f_{0}\cdot \;v_{0l}/c$ is the Doppler frequency, $f_{rl}=\;2\mu R_{0l}/c$ is related to the initial distance of the moving target, and $T_{s}\;=\;1/{\;f}_{s}$ is the sampling interval. Since $f_{rl}\gg f_{dl}$, the impact of the Doppler frequency on the fast-time dimension processing is very small, allowing us to ignore it and obtain sufficient range information.

Thus, from (9) and (10) we can derive the following expressions:

(11) $$R_{0l}\;=\;\frac{{\;c\;f}_{s}}{2\mu N}\;\cdot \;\gamma_{l}\;=\;\frac{c}{2\mu }\;\cdot \;f_{rl}$$

(12) $$v_{0l}=\frac{c}{2f_{0}M\cdot T_{l}}\cdot \;\vartheta_{l}=\frac{\lambda }{2}\;\cdot \;f_{dl}$$

From the above processing process of 2D-FFT algorithm, the target’s range and velocity can be estimated. Equations (11) and (12) are the expressions for the target’s estimated range and velocity. Therefore, the steps for the 2D-FFT algorithm for joint range–velocity estimation are shown in Algorithm 1:
**Algorithm 1** The 2D-FFT algorithm for joint range–velocity estimation**Require:** The IFS x(n, m) The number of sampling points *N* and chirps *M* **Ensure:** the range and velocity between target(s) and radar
Input the IFS x(n, m)
Perform an N-point FFT on the x(n, m) in the fast-time dimension, as shown in (7), and obtain the target range bin through peak detection. Perform an FFT for *M* chirps in the slow-time dimension as shown in (8), and obtain the velocity bin through peak search. The range and velocity of the l-th target can be obtained based on the range bin and velocity bin, respectively, as described in (9)–(12).

#### 2.2.2. FFT-MUSIC Algorithm for Joint Range–Velocity Estimation

The FFT-MUSIC algorithm [20] combines the FFT [30] and the classic MUSIC algorithm [31,32,33,34] to estimate the target’s range and Doppler frequency. First, after performing the range FFT on the IFS x(n, m), the output expression of the range FFT result for the m-th chirp is given by (7). After the range FFT, the peak detection is performed to select the range cell corresponding to the target. The index value of the target’s range cell can be expressed as $I\;=\left[I_{1},I_{2},\cdots I_{l},\cdots ,I_{L}\right]$. Thus, the range FFT result for the l-th target is

(13) $$X_{rl}=\left[{\;x}_{r}(I_{l},\;0),{\;x}_{r}(I_{l},\;1),\;\cdots \;,{\;x}_{r}(I_{l},\;M-1)\right]$$

Next, $X_{rl}$ is used as the input of the MUSIC algorithm for Doppler estimation. The correlation matrix C of $X_{rl}$ is expressed as

(14) $$C=X_{rl}{X_{rl}}^{H}$$

where ${\left(\cdot \right)}^{H}$ denotes the Hermitian operator. To determine the noise subspace for MUSIC, the eigenvalue decomposition (EVD) is applied to the correlation matrix *C*, which yields the signal subspace $E_{S}$ and the noise subspace $E_{N}$.

(15) $$R_{X}=E\;\left[X_{rl}{X_{rl}}^{H}\right]=U\Lambda U^{H}=\left[\begin{array}{cc} E_{S} & E_{N} \end{array}\right]\left[\begin{array}{cc} \sum_{S} & 0\\ 0 & \sum_{N} \end{array}\right]\left[\begin{array}{c} E_{S}\\ E_{N} \end{array}\right]$$

where U is the matrix of eigenvectors and Λ is the matrix of eigenvalues, with $\sum_{S}\;=\;diag(\lambda_{0},\;\lambda_{1},\;\cdots ,\;\lambda_{L-1})$ and $\sum_{N}\;=\;diag(\lambda_{L},\;\lambda_{L+1},\;\cdots ,{\;\lambda }_{M-1})$ representing the signal eigenvalues and noise eigenvalues, respectively.

Since the signal subspace $E_{S}$ and the noise subspace $E_{N}$ are orthogonal, we obtain

(16) $$a^{H}(\omega )E_{N}=0\;,\;\omega =\omega_{1},\;\omega_{2},\;{\cdots ,\;\omega }_{L}$$

where $a(\omega )\;=\;{\left[1,\;exp(-j\omega ),\;\cdots ,\;exp(-j\omega (M-1))\right]}^{T}$ with $\omega =2\pi f_{dl}T_{l}$. We can then construct the polynomial given by

(17) $$f(\omega )=a^{H}(\omega )E_{N}{E_{N}}^{H}a(\omega )$$

Since the signal vector a(ω) and the noise subspace are orthogonal, for the signal frequency $\omega_{l}$, we have $\;f(\omega_{l})\;=\;0$.

Finally, the MUSIC spectrum used for Doppler estimation is obtained.

(18) $$S_{MUSIC}(\omega )=\frac{1}{f(\omega )}=\frac{1}{a^{H}(\omega )E_{N}{E_{N}}^{H}a(\omega )}$$

By identifying the peaks of the above MUSIC spectrum  S(ω), the frequency $\omega_{l}$ can be determined, which is then used to calculate the velocity. Therefore, the velocity estimation expression of the MUSIC algorithm is given by

(19) $${\hat{v}}_{l}=\frac{\lambda }{2T_{l}}\omega_{l},\;\;l=1,\;2,\;\ldots ,\;L$$

In conclusion, the FFT-MUSIC algorithm for joint range–velocity estimation is detailed in Algorithm 2, which comprehensively illustrates the sequential steps of the proposed approach.
**Algorithm 2** The FFT-MUSIC algorithm for joint range–velocity estimation**Require:** The IFS x(n, m) The number of sampling points N and chirps M **Ensure:** the range and velocity between target(s) and radar
Input the IFS x(n, m)Perform range FFT and obtain the range cell and its corresponding range estimation frequency value.Use the range FFT result for the l-th target as the input of MUSIC, as illustrated in (13).Apply the MUSIC algorithm to acquire the velocity bin corresponding to the peak frequency $\;\omega_{l}$, as illustrated in (14)–(18).The range and velocity of the l-th target can be derived based on the range estimation frequency and the velocity estimation frequency, respectively.

The 2D-FFT algorithm is simple and easy to implement, but it suffers from issues such as aliasing, spectral leakage, sidelobe effects, and grating lobes. These issues lead to significant errors in amplitude, frequency, and phase estimation, making it inadequate for practical applications. The FFT-MUSIC algorithm, in contrast, separates signal and noise subspaces by calculating eigenvalues and eigenvectors, and constructs a pseudospectrum using the orthogonality of the noise subspace. This ultimately enables precise estimation of signal source frequencies or directions. Compared to the 2D-FFT algorithm, the FFT-MUSIC algorithm improves estimation accuracy and can effectively distinguish multiple closely spaced targets. However, the FFT-MUSIC algorithm has greater computational complexity. Considering the limitations of existing algorithms, it is necessary to further investigate the appropriate algorithm or make corresponding improvements to the existing algorithms based on the specific application requirements.

## 3. Proposed CEEMD-SVD-FRM Algorithm for Joint Range and Velocity Estimation

In this section, the CEEMD-SVD-FRM algorithm for joint range and velocity estimation is described in detail. This algorithm utilizes the CEEMD-SVD method to reconstruct the beat signal for denoising, and then applies the FFT-Root-MUSIC algorithm to the reconstructed beat signal for joint range and velocity estimation.

The denoising process using the CEEMD-SVD method is as follows. First, the IFS *x*(*n, m*) in (6), which contains noise components, is used as the original signal for denoising. A new signal is constructed by repeatedly adding pairs of positive and negative additive Gaussian white noise *n*(*t*) with an equal amplitude of $\xi_{0}$ to the original signal, as follows:

(20) $$x_{i}^{p}(n,\;m)\;=\;x(n,\;m)\;+{{\;(-1)}^{p}\xi }_{0}n_{i}(n,\;m),\;i\;=\;1,\;2,\;\ldots ,\;K/2$$

where *p* is the coefficient controlling the sign of the noise, and $\xi_{0}$ is generated by multiplying a standard normal random variable N(0,1) by the parameter $N_{std}$. The $N_{std}$ represents the ratio of the noise standard deviation to the standard deviation of the original signal, and it controls the noise strength relative to the original signal. And K/2 is the number of noise additions, where K is the total number of noise-added signals, as each addition includes two signals controlled by p.

Next, the EMD is applied to the new signal $x_{i}^{p}(n,\;m)$, which includes the i-th added Gaussian white noise, yielding several IMFs ${imf}_{i,j}(n,\;m)$ and a residual component ${res}_{i}\left(n,\;m\right)$.

(21) $$x_{i}^{p}(n,\;m)=x(n,\;m)+{{\;(-1)}^{p}\xi }_{0}n_{i}(n,\;m)=\sum_{j=1}^{D}{imf}_{i,j}(n,\;m)+{\;res}_{i}(n,\;m)$$

where j  denotes the layer number of the IMF components obtained from the decomposition of the signal $x_{i}(n,\;m)$, j=1, 2, ⋯, D.

Subsequently, for each input signal $x_{i}^{p}(n,\;m)$, the signal is decomposed to obtain K/2 IMFs, denoted as ${{imf}_{i,j}}^{\pm }(n,\;m)$. Compute the average of these IMFs to obtain the j-th IMF component of the ensemble empirical mode decomposition (EEMD), denoted as ${\overset{-}{imf}}_{j}(n,\;m)$:

(22) $${\overset{-}{imf}}_{j}\left(n,\;m\right)\;=\;\frac{1}{K}\sum_{i=1}^{\frac{K}{2}}{{imf}_{i,j}}^{\pm }\left(n,\;m\right)$$

Thus, during this process, the overall reconstruction error of the CEEMD is given by

(23) $$\varphi_{CEEMD}\left(n,\;m\right)=-\frac{1}{K}\sum_{i=1}^{\frac{K}{2}}\left[{\;\xi }_{0}n_{i}(n,\;m)+(-\xi_{0})n_{i}(n,\;m)\right]=0$$

**Figure 2:**
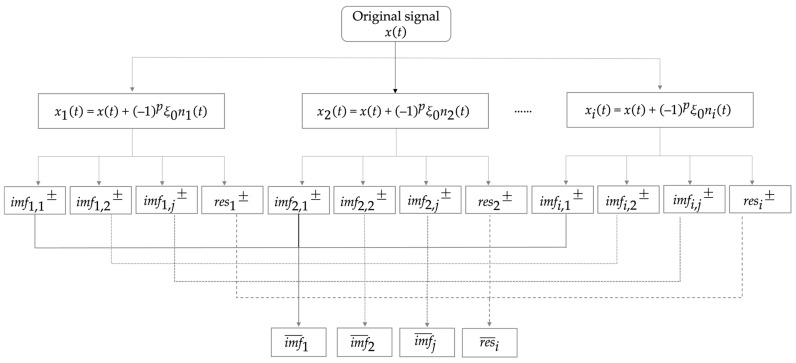
Flowchart of the CEEMD process.

From (23), it can be observed that the reconstruction error of the CEEMD is zero, effectively resolving the reconstruction error issue encountered in the EEMD during the processing. The flowchart of the CEEMD is illustrated in Figure 2.

In the actual processing, the original signal is the  x(n, m) in (6). After adding Gaussian white noise G times (where G= K/2) to the original signal, each chirp of the signal  x(n, m) is decomposed, and the obtained D IMFs can be expressed as in (24), along with a residual component ${\overset{-}{res}}_{G}(n,\;m)$.

(24) $$\;Y_{G}(n,\;m)={\left[{\overset{-}{imf}}_{1}(n,\;m),\cdots ,\;{\overset{-}{imf}}_{j}(n,\;m),\cdots ,{\overset{-}{imf}}_{D}(n,\;m)\right]}^{T}$$

Although CEEMD mitigates the interference caused by adding a single Gaussian white noise in EMD by adding paired positive and negative Gaussian white noise, which cancels out during decomposition, the residual noise still exists and can affect the signal. Moreover, this residual noise tends to transfer from high-frequency IMFs to low-frequency IMFs. Therefore, in the proposed CEEMD-SVD method, the SVD method is proposed to be utilized during the CEEMD process to further denoise and reduce the impact of residual noise on the signal.

After obtaining the IMFs and residuals through the CEEMD, the correlation between each IMF component and the original signal is used to indicate the degree of relationship between these two variables [35]. The D IMF components obtained through the CEEMD are denoted as $Y_{G}(n,\;m)$, as shown in (24). So, the correlation coefficient between each IMF component and the original signal is expressed as

(25) $$r_{j}=\frac{Cov\left(x(n,\;m),\;{\overset{-}{imf}}_{j}(n,\;m)\right)}{\sigma_{x}{\;\sigma }_{{\overset{-}{imf}}_{j}}}=\frac{E\left\{\left[x(n,\;m)-\;E\left(x(n,\;m)\right)\right]\left[{\overset{-}{imf}}_{j}(n,\;m)-\;E\left({\overset{-}{imf}}_{j}(n,\;m)\right)\right]\right\}}{\sqrt{E\left\{{\left[x(n,\;m)\right]}^{2}\right\}-{\left\{E\left[x(n,\;m)\right]\right\}}^{2}}\sqrt{E\left\{{\left[{\overset{-}{imf}}_{j}(n,\;m)\right]}^{2}\right\}-{\left\{E\left[{\overset{-}{imf}}_{j}(n,\;m)\right]\right\}}^{2}}}$$

where x(n, m) denotes the original signal, $Cov(x(n,\;m),{\overset{-}{imf}}_{j}(n,\;m))$ represents the covariance between x(n, m) and ${\overset{-}{imf}}_{j}(n,m)$, and $\sigma_{x}$ and ${\;\sigma }_{Y_{G}}$ are their respective standard deviations. $E\left(x(n,\;m)\right)$ and $E({\overset{-}{imf}}_{j}(n,m))$ denote their respective means. The correlation coefficient ranges from [−1, 1]. The larger the absolute value of the correlation coefficient, the stronger the relationship between the variables.

For the D IMFs obtained through CEEMD, their correlation with the original signal is calculated using (25). An appropriate threshold is applied to select k IMFs that are highly correlated with the original signal. These components can be represented by a N × k two-dimensional matrix, where *N* represents the number of sampling points. Thus, the k IMFs can be reconstructed into signal Y and its norm is

(26) $${\left\|\left.Y\right\|\right.}_{F}=\sqrt{\sum_{n=1}^{N}\sum_{m=1}^{k}{Y_{G}(n,m)}^{2}}$$

The total energy of the signal can be represented as

(27) $${{\left\|\left.Y\right\|\right.}_{F}}^{2}=trace\left\{YY^{T}\right\}=trace\left\{Y^{T}Y\right\}$$

where *trace*{ } denotes the trace of the matrix, which is equal to the sum of its diagonal elements.

Under the condition N>k, the rank of the matrix Y is k. By selecting the top P singular values for matrix reconstruction, the reconstructed matrix is denoted as $Y_{P}$ and can be expressed as

(28) $$Y_{P}=U\sum_{P}V^{T}$$

where ∑ represents a diagonal matrix, with $\sum_{P}\;=\;diag(\sigma_{1},\;\sigma_{2},\;\ldots \;,\;\sigma_{P},\;0,\;\ldots ,\;0)$, i.e., 1 < P <Q. U and V are orthogonal matrices formed by the eigenvectors of $YY^{T}$ and $Y^{T}Y$, respectively.

From (27) and (28), the following can be obtained:

(29) $${{\left\|\left.Y\;-\;Y_{P}\right\|\right.}_{F}}^{2}={{\left\|\left.U\sum V^{T}\;-\;U\sum_{P}V^{T}\right\|\right.}_{F}}^{2}=\sum_{i=P+1}^{Q}\sigma_{i}^{2}$$

Obviously, we have

(30) $${{\left\|\left.Y\right\|\right.}_{F}}^{2}=\sum_{i=1}^{k}\sigma_{i}^{2}$$

As can be seen from (30), the total energy of the signal is equal to the sum of the diagonal elements of the matrix, which corresponds to the sum of the non-zero singular values. During the signal reconstruction, larger singular values indicate stronger signal energy and make a more significant contribution to the reconstruction.

In summary, the process of the CEEMD-SVD joint denoising method is as follows: First, D IMFs obtained through CEEMD by adding noise G times, as shown in (24). Then, using (25), the correlation between each IMF and the original signal is calculated. The k highly correlated IMFs are selected and reconstructed into Y, as given in (26). Next, Y is decomposed using SVD, and the top P singular values are chosen for signal reconstruction, as shown in (28). Finally, the components $Y_{P}$ in (28) and a residual ${\overset{-}{res}}_{G}(n,\;m)$ obtained after the CEEMD-SVD method are utilized to obtain the reconstructed signal $\hat{Y}(n,\;m)$, where 0 ≤ n ≤ N−1, 0 ≤ m ≤ M−1.

(31) $$\;\hat{Y}(n,\;m)=Y_{P}(n,\;m)+{\overset{-}{res}}_{G}(n,\;m)$$

Then, the FFT-Root-MUSIC algorithm is applied to the reconstructed signal $\hat{Y}(n,\;m$) for joint range and velocity estimation. In contrast to the standard MUSIC algorithm, the Root-MUSIC eliminates the requirement for spectral peak searching, thus significantly reducing computational complexity.

Firstly, an N-point FFT is performed on the reconstructed signal $\hat{Y}(n,\;m$) to obtain the range frequency domain signal ${\hat{Y}}_{r}(\gamma ,\;m)$, similar to (7). Then, the peak detection is then applied to ${\hat{Y}}_{r}(\gamma ,\;m)$ to identify the range peak index $\;I_{l}$ for the l-th target. The peak frequency of the l-th target, i.e., its range frequency $f_{r(l)}\;$, is determined by the peak index $I_{l}$ and the frequency resolution ∆f as follows:

(32) $$f_{r(l)}\;={\;I}_{l}\;\cdot \;∆f,\;\;l\;=\;1,\;2,\;\ldots ,\;L$$

where the frequency resolution $∆f=\;f_{s}/N$. Then, the range of the detected l-th target is estimated using the (11).

Next, all range units corresponding to each target [20] are expressed as

(33) $$R_{bins}=\left[\begin{array}{cc} {\hat{Y}}_{r}(I_{1},0) & \begin{array}{ccc} {\hat{Y}}_{r}(I_{1},1) & \ldots & {\hat{Y}}_{r}(I_{1},M-1) \end{array}\\ {\hat{Y}}_{r}(I_{l},0) & \begin{array}{ccc} {\hat{Y}}_{r}(I_{l},1) & \ldots & {\hat{Y}}_{r}(I_{l},M-1) \end{array}\\ \begin{array}{c} \vdots \\ {\hat{Y}}_{r}(I_{L},0) \end{array} & \begin{array}{ccc} \begin{array}{c} \vdots \\ {\hat{Y}}_{r}(I_{L},1) \end{array} & \begin{array}{c} \ldots \\ \ldots \end{array} & \begin{array}{c} \vdots \\ {\hat{Y}}_{r}(I_{L},M-1) \end{array} \end{array} \end{array}\right],\;l=1,\;\ldots ,\;L$$

The l-th target is selected as the Doppler input, that is to say, the range FFT result $R_{bins,I_{l}}$ of the l-th target is set as the input signal for the Root-MUSIC algorithm.

(34) $$\;R_{bins,I_{l}}=\left[\begin{array}{ccc} {\hat{Y}}_{r}(I_{1},0) & {\hat{Y}}_{r}(I_{1},1) & \begin{array}{cc} \cdots & {\hat{Y}}_{r}(I_{1},M-1) \end{array} \end{array}\right]$$

Next, $R_{bins,I_{l}}$ is submitted into (14) to obtain the correlation matrix, which is used by the Root-MUSIC algorithm for Doppler frequency estimation. To determine the noise subspace for the Root-MUSIC, we apply the EVD to the correlation matrix, as shown in (15), which yields the signal subspace $G_{S}$ and the noise subspace $G_{N}$.

(35) $$R_{\hat{Y}}=E\;\left[R_{bins,I_{l}}{R_{bins,I_{l}}}^{H}\right]=\left[\begin{array}{cc} G_{S} & G_{N} \end{array}\right]\left[\begin{array}{cc} \sum_{S} & 0\\ 0 & \sum_{N} \end{array}\right]\left[\begin{array}{c} G_{S}\\ G_{N} \end{array}\right]$$

Then, the Root-MUSIC algorithm treats $e^{jw}$ in the MUSIC algorithm‘s a(ω) as a complex variable z, thus yielding

(36) $$f(z)=p^{H}(z)G_{N}{G_{N}}^{H}p(z)=0$$

where $p(z)={\left[1,\;z,\cdots ,\;z^{M-1}\right]}^{T}$,$z=e^{j\omega }$. The estimation of signal frequencies is thereby transformed from a search or exhaustive scanning problem into a root-finding problem for a univariate high-degree polynomial. Since we are only interested in the z-values closest to the unit circle, (36) can be transformed into

(37) $$f(z)\;=z^{M-1}p^{T}(z^{-1})G_{N}{G_{N}}^{H}p(z)=0$$

Equation (36) has a total of 2(M−1) roots, but only the L roots located on the unit circle are the desired solutions. Then, the Doppler frequency is estimated as

(38) $$f_{d(l)}=\frac{z_{l}}{2\pi T_{l}},\;l=1,\;\ldots ,\;L$$

Based on the frequency estimate $f_{peak,{\;I}_{l}}$ for the range dimension and $f_{l}$ for the Doppler dimension, the target’s distance and speed are estimated as follows:

(39) $$\hat{R_{l}}\;=\;\frac{c}{2\mu }\cdot f_{peak,I_{l}}$$

(40) $$\hat{V_{l}}=\frac{\lambda }{2}\cdot \;f_{l}$$

To summarize, the CEEMD-SVD-FRM algorithm for joint range and velocity estimation was summarized in Algorithm 3, which systematically presents the procedural steps of the proposed method.
**Algorithm 3** The CEEMD-SVD-FRM algorithm for joint range and velocity estimation**Require:** The IFS x(n, m) The number of the target L The number of sampling points N and chirps M **Ensure:** the range and velocity between target(s) and radar 
Perform the CEEMD on x(n, m) with G iterations of added noise to acquire D IMFs and a residual component.Directly discard the first-order IMF component.Calculate the correlation between the D$\;\mathrm{IMFs}\;Y_{G}(n,\;m)$ and the original signal x(n, m).Select k highly correlated IMFs for the SVD denoising, and use the top P largest singular values for signal reconstruction,Select the top P largest singular values for signal reconstruction, as seen in (28).$\mathrm{Reconstruct}\;\mathrm{the}\;\mathrm{signal}\;\hat{Y}(n,\;m$).$\mathrm{Perform}\;\mathrm{the}\;\mathrm{range}\;\mathrm{FFT}\;\mathrm{on}\;\hat{Y}(n,\;m)$$\;\mathrm{to}\;\mathrm{obtained}\;f_{r(l)}$$\;\mathrm{and}\;R_{bins}$ in (32) and (33), l=1, …,L.Select the l-th$\;target\;\mathrm{FFT}\;\mathrm{result}\;R_{bins,I_{l}}\;$$\mathrm{as}\;\mathrm{the}\;\mathrm{input}\;\mathrm{signal}\;\mathrm{for}\;\mathrm{the}\;\mathrm{Root}\text{-}MUSIC\;\mathrm{algorithm},\;\mathrm{to}\;\mathrm{obtain}\;\mathrm{the}\;\mathrm{Doppler}\;\mathrm{frequency}\;\mathrm{estimate}\;f_{d(l)}$, l=1, …,L.$\mathrm{Calculate}\;\hat{R_{l}}\;$, the range estimation of the l-th target, as presented in (39)$\mathrm{Calculate}\;\hat{V_{l}}$, the velocity estimation of the l-th target, as presented in (40)

## 4. Computational Complexity Analysis

In this section, the computational complexity of the proposed CEEMD-SVD-FRM algorithm is analyzed, using multiplication operations as the benchmark for complexity evaluation, since multiplication is generally more computationally intensive compared to other operations such as additions.

In the CEEMD-SVD-FRM algorithm, a signal with M chirps and N sampling points is first decomposed using CEEMD. After adding Gaussian noise G (where G = K/2) times and applying EMD, D IMFs are obtained through weighted averaging of the K IMF components from j-th layer. These IMF components, denoted as $Y_{G}(n,\;m)$, are then denoised IMFs using SVD, where the top P largest singular values are selected for signal reconstructions. The denoised IMFs and the residual signal are reconstructed, followed by range FFT and velocity estimation using the Root-MUSIC algorithm. Thus, the complexity of the CEEMD involves K iterations for each layer, where each iteration processes N samples. The complexity of the CEEMD step is $O\left(KMN\right)$. After the CEEMD, calculating the correlation coefficient between each IMF component and the original signal requires $O\left(KDMN\right)$ additions and multiplications. For k high-correlation IMFs, the SVD complexity is $\;O\left(k^{2}N\right)$ for additions and $\;O\left(k^{3}\right)$ for multiplications. The complexity of one-dimensional FFT is $\;O\left(NlogN\right)$ for both additions and multiplications, and the complexity of the Root-MUSIC algorithm is approximately $\;O\left(M^{2}+M^{3}\right)$ for both additions and multiplications. By focusing on the highest order of complexity, the primary influencing factors are simplified, and a complexities analysis of the computational complexities of the proposed algorithm, 2D-FFT [9,10], 2D-CZT (Chirp-Z Transform) [36], 2D-MUSIC [22,37,38], FFT-MUSIC [20], and FFT-Root-MUSIC algorithms is conducted in terms of multiplications operation, as illustrated in Table 2.

According to Table 2, the proposed CEEMD-SVD-FRM algorithm for joint range and velocity estimation exhibits a computational complexity that is higher than that of the FFT-Root-MUSIC algorithm. Compared to the 2D-FFT, 2D-CZT, and FFT-MUSIC algorithms, its complexity is also greater, although it remains lower than that of the 2D-MUSIC algorithm. Notably, the CEEMD-SVD-FRM algorithm demonstrates superior resolution, estimation accuracy, and robustness in high-noise environments compared to the other four algorithms. Therefore, although there is an increase in computational complexity compared to traditional range and velocity estimation algorithms, the CEEMD-SVD-FRM algorithm still offers significant advantages, particularly in maintaining high resolution, high estimation accuracy, and strong robustness under low SNR conditions.

## 5. Simulations and Analysis

In this section, the performance of the proposed CEEMD-SVD-FRM algorithm is verified through simulation studies and field test results for joint range and velocity estimation in the FMCW radar. The parameters for both the simulation studies and field test are provided in Table 3. In the simulation experiments, the performance of the CEEMD-SVD-FRM algorithm was compared with that of the 2D-FFT, 2D-CZT, 2D-MUSIC, FFT-MUSIC, and FFT-Root-MUSIC algorithms. Monte Carlo simulations were performed to calculate the root mean square error (RMSE) of the range and velocity estimation results, and all results were obtained from over 100 independent Monte Carlo trials.

### 5.1. Simulation Studies

Based on the parameter settings in Table 3, the MATLAB (MathWorks, Natick, USA; version R2021b) was used to implement the 2D-FFT, 2D-CZT, 2D-MUSIC, FFT-MUSIC, FFT-Root-MUSIC, and the proposed CEEMD-SVD-FRM algorithms. In total, 100 Monte Carlo simulations were conducted to calculate the mean and RMSE of the range and velocity estimation results.

According to the parameter settings in Table 3, the maximum range is 200 m, the maximum velocity is 3.8344 m per second, the range resolution is 0.1667 m, and the velocity resolution is 0.2396 m per second. Since the range estimation accuracy for the algorithms discussed in this section falls between 40 mm and 160 mm, further elaboration on range estimation will not be provided. This section will focus on comparing the RMSE of velocity estimation for each algorithm and analyzing the performance of joint range–velocity estimation across the algorithms.

#### 5.1.1. Estimation Performance for a Single-Target Scenario

For the single-target simulation at a range of 2.5 m and a velocity of 0.8 m/s, 100 Monte Carlo simulations were conducted to compute the RMSE for range and velocity estimations, as well as to record the average execution time per run of the various algorithms.

**Figure 3:**
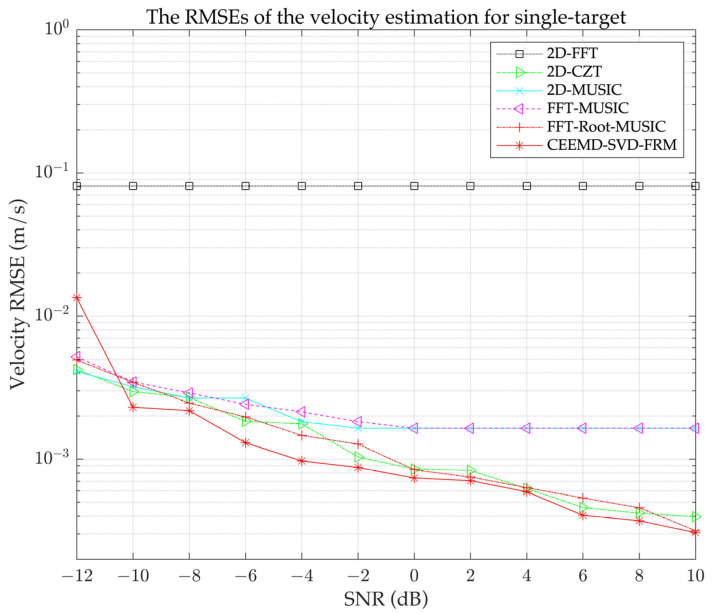
The RMSE of single-target velocity estimation for different SNR levels.

The RMSE results for velocity estimation are illustrated in Figure 3. As can be seen in Figure 3, the 2D-FFT exhibits the worst performance due to the spectral leakage effect. The 2D-CZT, which is optimized based on FFT, demonstrates much better performance compared with the 2D-FFT, 2D-MUSIC, and FFT-MUSIC, especially as the SNR increases. Overall, 2D-MUSIC and FFT-MUSIC algorithms show similar performance, but the computational complexity of the FFT-MUSIC algorithm is much lower than that of the 2D-MUSIC algorithm. In contrast, the proposed CEEMD-SVD-FRM algorithm performs the best among these algorithms. This is mainly because we introduced a CEEMD-SVD joint denoising preprocessing method into the FFT-Root-MUSIC algorithm, which significantly improves signal quality and mitigates the effect of noise on the estimation performance of the FFT-Root-MUSIC algorithm.

The average execution time per run (calculated from multiple experiments) for each algorithm is summarized in Table 4. Table 4 demonstrates that the CEEMD-SVD-FRM algorithm has a lower average execution time than the 2D-MUSIC, but higher than other algorithms. So, it offers a better trade-off between the estimation accuracy and the computational efficiency, and thus is more suitable for practical applications.

#### 5.1.2. Estimation Performance for a Multi-Target Scenario

For the multi-target scenario, with initial ranges of 2.5 m and velocities of 0.3 m/s and 0.7 m/s, 100 Monte Carlo simulations were conducted to compute the RMSE of the range and velocity estimations, as well as to record the execution times of the algorithms.

**Figure 4:**
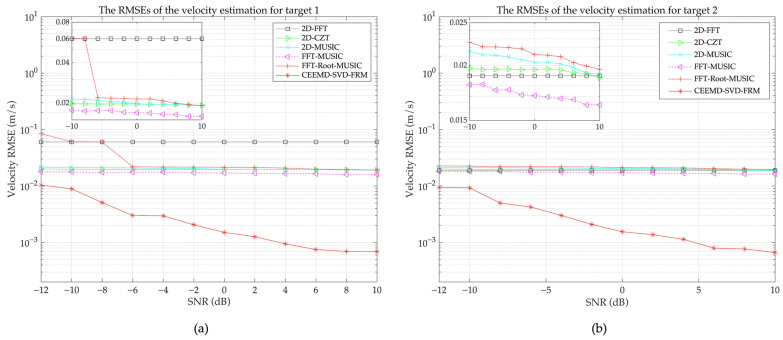
The velocity RMSE of various algorithms under different SNR levels. (**a**) The RMSEs of the velocity estimation for target 1; (**b**) The RMSEs of the velocity estimation for target 2.

The RMSEs of the velocity estimation of various algorithms under different SNR levels are depicted in Figure 4. As illustrated in Figure 4, the FFT algorithm is limited by its fixed frequency resolution and spectral leakage, and thus its performance is worst among these algorithms. The 2D-CZT shows improved RMSE performance compared to 2D-FFT at lower SNR levels. Subspace-based algorithms, such as 2D-MUSIC and FFT-MUSIC, exhibit a gradual decrease in RMSE with increasing SNR, leading to improved estimation performance. In contrast, the proposed CEEMD-SVD-FRM algorithm achieves the lowest velocity RMSE across all SNR conditions, demonstrating higher velocity estimation accuracy and higher resolution than other algorithms.

Table 5 illustrates the average execution time per run (calculated from multiple experiments) of various algorithms in the multi-target scenario, which is used to evaluate the computational efficiency of each algorithm. As can be seen from Figure 4 and Table 5, the CEEMD-SVD-FRM algorithm achieves superior accuracy with reasonable computational efficiency. These results highlight that the CEEMD-SVD-FRM algorithm offers a better trade-off between the precision and the computational efficiency, and thus is more suitable for practical applications.

### 5.2. Field Test Results

#### 5.2.1. The Rail Teat System for FMCW Radar

**Figure 5:**
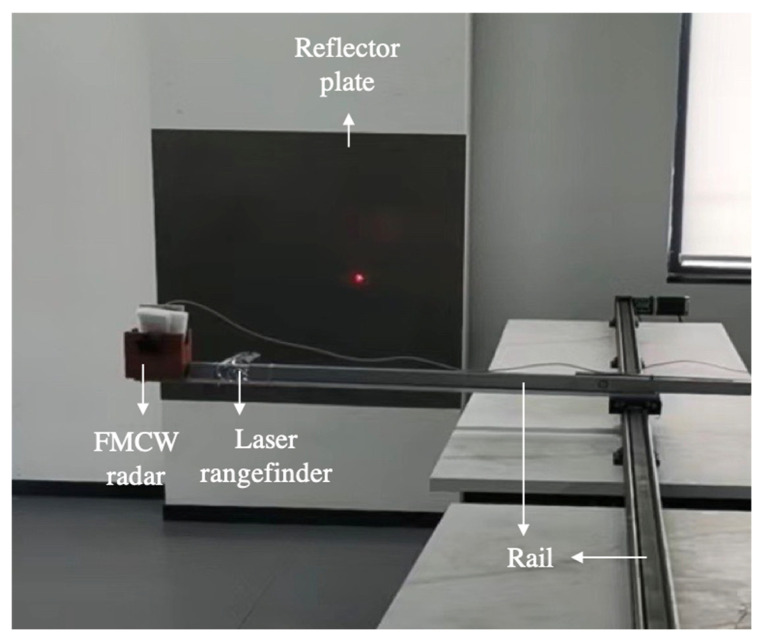
The rail test system for FMCW radar.

In this section, a rail test system for the FMCW radar was built to verify the performance of the proposed algorithm in real-world engineering applications [39]. As shown in Figure 5, the test system includes an FMCW radar, a laser rangefinder, a reflector plate mounted on the wall, and a rail for mounting the FMCW radar. In this setup, the FMCW radar emits signals, which are then reflected back by the reflector plate. The laser rangefinder measures the reference distance between the radar and the reflector plate, while the rail system is used to fix or automatically adjust the distance between them. The distance and velocity information of the measured target is derived from the beat frequency signals received at various distances between the radar and the reflector plate within this measurement system [40]. This FMCW radar measurement system is utilized to validate the performance of the proposed CEEMD-SVD-FRM algorithm under real-world conditions. The relevant parameters of the measurement system are detailed in Table 3.

Utilizing the above test system, the distance between the radar and the reflector is adjusted between 1500 mm and 3000 mm, and then the FMCW radar will move at a constant speed along the guide rail in this distance range to obtain the measurement data. Measurements were conducted at three initial speeds: stationary of 0 m/s, low speed of 0.11 m/s, medium speed of 0.41 m/s, and high speed of 0.8 m/s. For the measured signal, 1024 sampling points were obtained from 32 Chirps, and 100 Monte Carlo experiments were conducted to verify the velocity estimation performance of various algorithms for the case of a single target. Table 6, Table 7 and Table 8 give the mean velocity of various algorithms, the velocity RMSE of various algorithms, and the average execution time per run, respectively, in the rail test system for the FMCW radar.

As can be seen from Table 6 and Table 7, the mean velocity of the CEEMD-SVD-FRM algorithm is closer to the actual velocity compared to the other algorithms, and its RMSE for velocity estimates is lower than that of the other algorithms. Therefore, the CEEMD-SVD-FRM algorithm outperforms other methods in terms of estimation accuracy and can effectively improve the estimation accuracy of the FMCW radar system. From Table 8, it is evident that the computational efficiency of the CEEMD-SVD-FRM algorithm is reasonable, though it requires more processing time than the 2D-FFT and the FFT-MUSIC algorithm. This reflects that the CEEMD-SVD-FRM algorithm achieves a better trade-off between the computational cost and the accuracy among these algorithms. Therefore, the CEEMD-SVD-FRM algorithm is well suited for applications where higher accuracy is prioritized over the computational efficiency.

#### 5.2.2. The Conveyor Belt Test System for Mimicking Water Velocity Measurement by Utilizing the FMCW Radar

**Figure 6:**
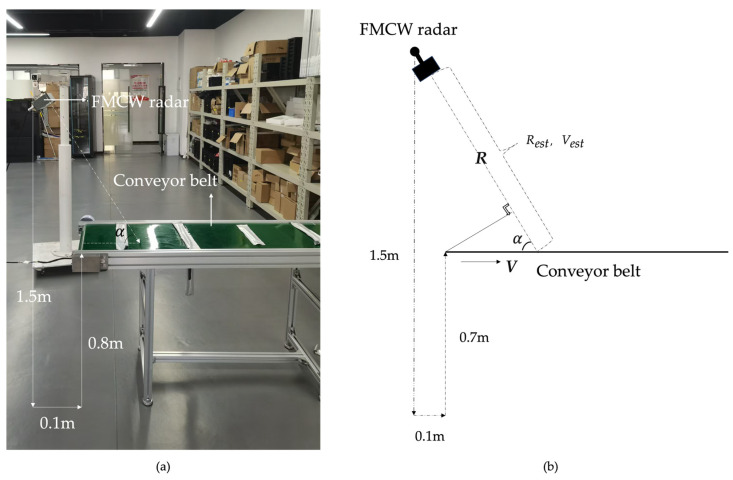
The conveyor belt test system for FMCW radar. (**a**) The front of the experimental scene; (**b**) Experimental setup parameter diagram.

To expand the experimental validation, we conducted an additional use scenario for the FMCW radar, as shown in Figure 6a, to strengthen the application case of the proposed algorithm. The conveyor test system for FMCW radar was constructed to mimic the scenario of the water velocity measurements by utilizing the FMCW radar. As demonstrated in Figure 6b, the FMCW radar is 1.8 m from the ground, the conveyor belt device is 0.8 m from the ground, the horizontal range between the radar and the conveyor belt device is 0.1 m, and the horizontal angle between the radar and the conveyor belt device is α. As can be seen from Figure 6b, the relation between the velocity of the conveyor belt movement V and the estimated radial velocity $V_{est}$ by the FMCW radar is expressed as follows:

(41) $$V\;=\;\frac{V_{est}}{cos(\alpha )}$$

Utilizing the conveyor belt test system for FMCW radar, the conveyor belt was set to move at four different speeds: 0 m/s, 0.24 m/s, 0.75 m/s, and 1.0 m/s. For each speed, the IFS of the FMCW radar was collected, and each frame of the collected data is composed of 32 chirps and each chirp has 1024 sampling points. Then, the collected data were processed by the CEEMD-SVD-FRM algorithm to obtain the velocity estimation results. Table 6 and Table 7 give the velocity RMSE of various algorithms and the average execution time per run, respectively, in the rail test system for the FMCW radar.

Based on the data from Table 9, Table 10 and Table 11, the CEEMD-SVD-FRM algorithm demonstrates significant advantages in the single-target scenario. From Table 9, the mean velocity estimation of the CEEMD-SVD-FRM is closest to the actual velocity. Then, the RMSE results in Table 10 further confirm this, with CEEMD-SVD-FRM exhibiting significantly lower RMSE across all test velocities. It is noteworthy that for a stationary target, both the mean velocity and RMSE estimated by 2D-FFT are zero. This is because the 2D-FFT algorithm, based on frequency-domain analysis, focuses the spectral components at the zero frequency point when the target velocity is zero, leading to an estimated value close to zero. Inaddition, the average execution time per run of CEEMD-SVD-FRM is relatively long, as seen in Table 11. Finally, as can be seen from Table 10 and Table 11, the proposed CEEMD-SVD-FRM algorithm offers the best accuracy with reasonable computational cost, making it an optimal choice for practical applications requiring precise velocity estimation.

## 6. Conclusions

To address the issues of low-estimation accuracy, poor resolution, and weak robustness in existing joint range and velocity estimation algorithms, this study proposes a novel approach that combines CEEMD, SVD, FFT, and the Root-MUSIC algorithm. This approach is based on FMCW radar and is referred to as the CEEMD-SVD-FRM algorithm for joint range and velocity estimation.

First, the IF signal, which is contaminated with Gaussian white noise, is processed using CEEMD. An appropriate autocorrelation coefficient threshold is then determined to select the IMFs with high correlation. Subsequently, the SVD is employed to denoise the selected high-correlation IMFs. The denoised IMFs and the residual signal are then combined to reconstruct the signal. Next, FFT is applied to extract the frequency domain signal and perform peak detection. The frequency peak index *I* is used to obtain the frequency estimate in the range dimension, and the result for the *l*th target (among L targets, *l* = 1, …, L) is selected as the input for subsequent signal processing. The Root-MUSIC algorithm is then used to obtain the frequency estimate in the Doppler dimension. Finally, the range and velocity estimates of the target are calculated using the frequency estimates from both the range and Doppler dimensions. Through several experiments, it has been verified that the CEEMD-SVD-FRM algorithm effectively removes noise, preventing the loss of signal characteristics that can occur when performing direct Fourier transforms. As a result, this algorithm significantly enhances the accuracy and robustness of joint range and velocity estimation for target resolution in low SNR environments.

In the future, we will focus on optimizing the CEEMD-SVD denoising process or incorporating other methods to reduce the computational complexity of the algorithm. This optimization will have practical significance for the application of algorithms based on EMD.

## Figures and Tables

**Table 1:** Common radar waveform types.

**Waveform**	**Modulation Type**	**Duty Cycle (%)**
Simple pulse	Rectangular amplitude modulation	0.01~1
Intra-pulse modulated pulse	Linear/nonlinear frequency-modulation	0.1~10
	intra-pulse phase coding	
Interrupted CW with high duty cycle	Rectangular amplitude modulation	30~50
	linear frequency modulation	
FMCW	Linear frequency modulation	100
	Sine wave frequency-modulation	
	phase coding	
CW	/	

**Table 2:** Comparison of computational complexity for different algorithms.

Algorithm	Multiplication Complexity
2D-FFT	$M\cdot N{log}_{2}N+M{log}_{2}M$
2D-CZT	$M\cdot N{log}_{2}N+2MN+(M+N){log}_{2}\left(M+N\right)$
2D-MUSIC	$N^{2}M+M^{2}N+N^{3}+M^{3}$
FFT-MUSIC	$N{log}_{2}N+M^{3}$
FFT-Root-MUSIC	$N{log}_{2}N+M^{2}N+M^{3}$
CEEMD-SVD-FRM	$KMN\;+\;KDMN\;+k^{2}N+k^{3}+N{log}_{2}N+M^{2}+M^{3}$

**Table 3:** Simulations parameters.

Parameters	Value
$\mathrm{Initial}\;\mathrm{frequency}\;f_{0}/\mathrm{GHz}$	24.45
Bandwidth B/MHz	900
Speed of electromagnetic waves c/(m/s)	$3\times 10^{8}$
$\mathrm{Modulation}\;\mathrm{period}\;T_{c}/\mu s$	750
$\mathrm{Inter}\text{-}\mathrm{pulse}\;\mathrm{interval}\;T_{z}/\mu s$	50
$\mathrm{Sampling}\;\mathrm{frequency}\;f_{s}$/kHz	1600
SNR/dB	−12~10
Slow-time domain spatial sampling points *M*	32
Fast-time domain spatial sampling points *N*	1024

**Table 4:** The average execution time per run of various algorithms for the single-target scenario.

Algorithm	2D-FFT	2D-CZT	2D-MUSIC	FFT-MUSIC	FFT-Root-MUSIC	CEEMD-SVD-FRM
Time/s	0.0128	0.1920	69.6833	0.1505	0.1317	10.6689

**Table 5:** The average execution time per run of various algorithms for the multi-target scenario.

Algorithm	2D-FFT	2D-CZT	2D-MUSIC	FFT-MUSIC	FFT-Root-MUSIC	CEEMD-SVD-FRM
Time/s	0.00667	0.01729	29.33953	0.23629	0.06878	10.12273

**Table 6:** The mean velocity of various algorithms for the single-target scenario.

Actual Velocity	2D-FFT	2D-CZT	2D-MUSIC	FFT-MUSIC	FFT-Root-MUSIC	CEEMD-SVD-FRM
0 m/s	0	0.003422	0.003700	0.009700	0.004017	0.002160
0.11 m/s	0.167760	0.118811	0.101200	0.120700	0.120150	0.114063
0.41 m/s	0.559200	0.397867	0.401100	0.397325	0.427325	0.408971
0.80 m/s	0.838700	0.780670	0.783400	0.784518	0.786600	0.786720

**Table 7:** The velocity RMSE of various algorithms for the single-target scenario.

Actual Velocity	2D-FFT	2D-CZT	2D-MUSIC	FFT-MUSIC	FFT-Root-MUSIC	CEEMD-SVD-FRM
0 m/s	0	0.005841	0.003700	0.007137	0.008017	0.002993
0.11 m/s	0.148656	0.009630	0.008800	0.036630	0.011570	0.006192
0.41 m/s	0.149200	0.007118	0.008900	0.014223	0.011803	0.006281
0.80 m/s	0.038700	0.026513	0.016600	0.027478	0.014698	0.013399

**Table 8:** The average execution time per run of various algorithms for the single-target scenario.

**Algorithm**	**2D-FFT**	**2D-CZT**	**2D-MUSIC**	**FFT-MUSIC**	**FFT-Root-MUSIC**	**CEEMD-SVD-FRM**
Time/s	0.0026	0.0113	187.9735	0.04675	0.04217	25.3233

**Table 9:** The velocity mean of various algorithms for the single-target scenario.

Actual Velocity	2D-FFT	2D-CZT	2D-MUSIC	FFT-MUSIC	FFT-Root-MUSIC	CEEMD-SVD-FRM
0.00 m/s	0	0.000521	0.003491	0.004057	0.000504	0.000220
0.24 m/s	0.226149	0.247610	0.229855	0.246910	0.248594	0.246126
0.75 m/s	0.452299	0.756307	0.760426	0.756393	0.757133	0.755312
1.00 m/s	0.904517	1.021284	1.019815	1.021797	1.021095	0.998895

**Table 10:** The velocity RMSE of various algorithms for the single-target scenario.

Actual Velocity	2D-FFT	2D-CZT	2D-MUSIC	FFT-MUSIC	FFT-Root-MUSIC	CEEMD-SVD-FRM
0.00 m/s	0	0.003532	0.003491	0.004874	0.003681	0.003416
0.24 m/s	0.013851	0.023049	0.010145	0.022492	0.009218	0.007987
0.75 m/s	0.270654	0.051595	0.055900	0.051769	0.007498	0.006706
1.00 m/s	0.095483	0.021372	0.020088	0.082933	0.021182	0.018967

**Table 11:** The average execution time per run for various algorithms for the single-target scenario.

Algorithm	2D-FFT	2D-CZT	2D-MUSIC	FFT-MUSIC	FFT-Root-MUSIC	CEEMD-SVD-FRM
Time/s	0.0010	0.0026	134.3663	0.01847	0.0060	15.3622

## Data Availability

Data are contained within the article.

## Abbreviations

The following abbreviations are used in this manuscript:

FMCW	Frequency-modulated continuous wave
2D-FFT	Two-dimensional fast Fourier transform
FFT	Fast Fourier transform
SNR	Signal-to-noise ratio
ESPRIT	Estimating signal parameters via rotational invariance techniques
MUSIC	Multiple signal classification
CEEMD	Complementary ensemble empirical mode decomposition
SVD	Singular value decomposition
Root-MUSIC	Root multiple signal classification
EMD	Empirical mode decomposition
IFS	Intermediate frequency signal
IMF	Intrinsic mode function
EVD	Eigen value decomposition
EEMD	Ensemble empirical mode decomposition
CZT	Chirp-z transform
RMSE	Root mean square error
